# Cytological and Transcriptome Analyses Provide Insights into Persimmon Fruit Size Formation (*Diospyros kaki* Thunb.)

**DOI:** 10.3390/ijms25137238

**Published:** 2024-06-30

**Authors:** Huawei Li, Yujing Suo, Hui Li, Peng Sun, Shuzhan Li, Deyi Yuan, Weijuan Han, Jianmin Fu

**Affiliations:** 1 Key Laboratory of Cultivation and Protection for Non-Wood Forest Trees, Ministry of Education, Central South University of Forestry and Technology, No. 498 Shaoshan South Road, Changsha 410004, China; lihuaweicaf@163.com (H.L.); lishuzhan@163.com (S.L.); 2 Research Institute of Non-Timber Forestry, Chinese Academy of Forestry, No. 3 Weiwu Road, Jinshui District, Zhengzhou 450003, China; suoyj@caf.ac.cn (Y.S.); ptsunpeng@caf.ac.cn (P.S.); hanweijuan@caf.ac.cn (W.H.); 3 Research Institute of Forestry Policy and Information, Chinese Academy of Forestry, Xiangshan Road, Haidian District, Beijing 100091, China; lihui09610@163.com

**Keywords:** persimmon, fruit size, cell number, cell division, ethylene

## Abstract

Persimmon (*Diospyros kaki* Thunb.) fruit size variation is abundant. Studying the size of the persimmon fruit is helpful in improving its economic value. At present, the regulatory mechanism of persimmon fruit size formation is still unclear. In this study, the mechanism of fruit size formation was investigated through morphological, cytological and transcriptomic analyses, as well as exogenous ethrel and aminoethoxyinylglycine (AVG: ethylene inhibitor) experiments using the large fruit and small fruit of ‘Yaoxianwuhua’. The results showed that stages 3–4 (June 11–June 25) are the crucial morphological period for differentiation of large fruit and small fruit in persimmon. At this crucial morphological period, the cell number in large fruit was significantly more than that in small fruit, indicating that the difference in cell number is the main reason for the differentiation of persimmon fruit size. The difference in cell number was caused by cell division. *CNR1*, *ANT, LAC17* and *EB1C*, associated with cell division, may be involved in regulating persimmon fruit size. Exogenous ethrel resulted in a decrease in fruit weight, and AVG treatment had the opposite effect. In addition, *LAC17* and *ERF114* were upregulated after ethrel treatment. These results indicated that high ethylene levels can reduce persimmon fruit size, possibly by inhibiting cell division. This study provides valuable information for understanding the regulation mechanism of persimmon fruit size and lays a foundation for subsequent breeding and artificial regulation of fruit size.

## 1. Introduction

Persimmon (*Diospyros kaki* Thunb.) is one of the most popular fruits in China [1]. Fruit size, as the most intuitive external feature, is often the primary factor for consumers to consider when purchasing. In biology, fruit size is an important fitness trait in plant evolution. In the breeding process, fruit size, as a crucial agronomic trait for crop improvement, is the target of artificial selection [2,3]. In production, non-uniformity in fruit size affects mechanical harvesting and processing efficiency. The abundant diversity of persimmon fruit size laid a foundation for studying the regulation mechanism of fruit size, which is conducive to the subsequent artificial regulation of fruit size. 

Fruit size formation involves multiple biological processes, such as plant hormone biosynthesis and signal transduction, cell division, and cell expansion. Plant hormones are directly or indirectly involved in cell division and cell expansion during fruit development, ultimately affecting fruit size. In tomato (*Solanum lycopersicum*) and Arabidopsis (*Arabidopsis thaliana*), rapid accumulation of auxin in fertilized ovules stimulates cell division and cell expansion, thus promoting fruit growth [4,5]. *PINs* are involved in regulating the polar transport of auxin, and silencing *SlPIN4* reduces tomato fruit size [6]. In grape (*Vitis vinifera*), exogenous GA can promote cell division and increase fruit weight [7]. In jujube (*Ziziphus jujuba*), cytokinin controls fruit size by regulating cell division [8,9]. Low carbon levels directly inhibited the expression of cytokinin biosynthetic enzymes like *IPTs* and *CYP735As*, resulting in smaller fruit size of kiwifruit (*Actinidia chinensis*) [10]. In addition, ABA, ethylene and brassinosteroid have also been shown to be involved in regulating fruit size [11,12,13,14].

With the development of sequencing technology, more and more genes related to fruit size have been identified. For example, overexpression of *SlERF36* can reduce fruit size in tomato [14]. *ENO* regulates tomato fruit size, participating in floral meristem development [15]. *STK* encodes a MADS-box transcription factor, and *NTT* encodes a C2H2/C2HC zinc finger transcription factor; loss function in Arabidopsis leads to reduced silique length [16,17].

At present, research on persimmon fruit size is in its initial stage, and the regulatory mechanism of fruit size formation is still unclear. Here, the large fruit and small fruit of ‘Yaoxianwuhua’ were used as materials to carry out morphological, cytological, transcriptomic and exogenous hormone experimental studies. This study will provide valuable information for further exploring the mechanism of persimmon fruit size formation.

## 2. Results

### 2.1. Morphological Comparison of Fruit

**Figure 1:**
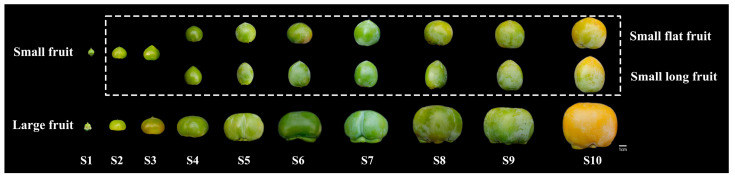
Morphological change in the ‘Yaoxianwuhua’ fruit. The figure in the white frame is from the following research ‘*Cytological, phytohormone, and transcriptome analyses provide insights into persimmon fruit shape formation* (*Diospyros kaki* Thunb.)’ [18].

**Figure 2:**
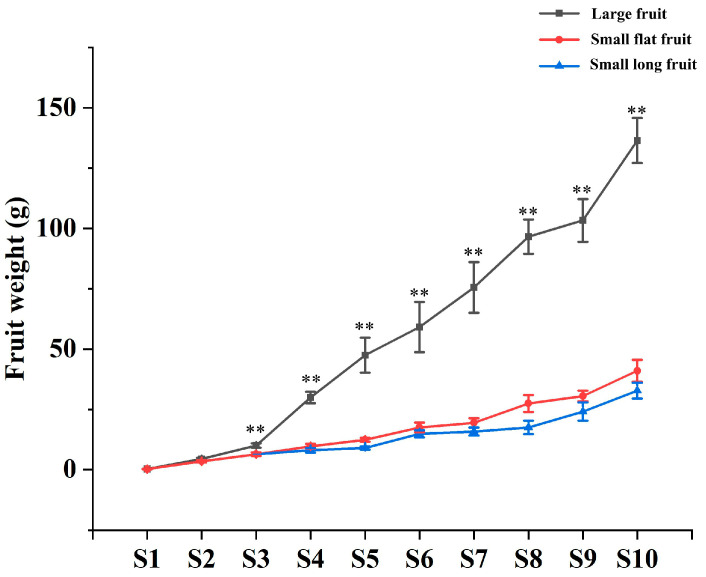
The difference of FW between LF and SF during fruit development. ** *p* < 0.01. In all figures, the note for ‘small fruit at stage 3’ was represented by ‘small flat fruit’.

The ‘Yaoxianwuhua’ persimmon is polygamomonoecious with a male flower, female flower and hermaphroditic flower. Interestingly, there is a significant difference in fruit size between female flower fruit and hermaphroditic flower fruit. Large fruit (LF) was produced by female flowers. Small fruit (SF), including small long fruit (SLF) and small flat fruit (SFF), were produced by hermaphroditic flowers. In order to study the reasons for the formation of different fruit sizes, we observed the phenotype of fruits throughout the development period. In this study, fruit size was measured by fruit weight (FW). In stages 1–2 (May 15–May 29), there was no significant difference in FW of LF and SF. In stage 3 (June 11), the FW began to differentiate, and the FW of LF was greater than that of SF. From stage 4 to stage 10 (June 25–September 15), SF differentiated into SFF and SLF. The FW of SF increased slightly, while the FW of LF increased substantially (Figure 1 and Figure 2). Therefore, stages 3–4 (June 11–June 25) are crucial for the morphological differentiation of LF and SF in persimmon.

### 2.2. Cytological Observation

**Figure 3:**
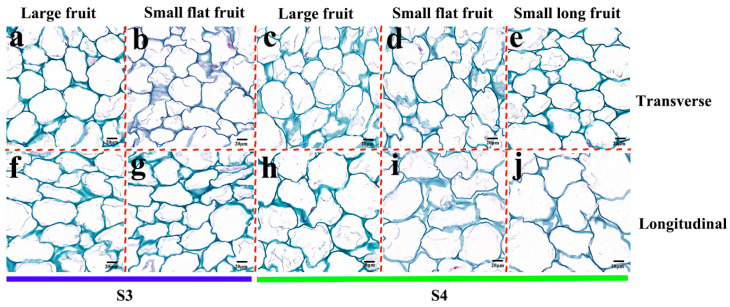
Cytological observations of fruit. Transverse section: (**a**) LF in stage 3; (**b**) SFF in stage 3; (**c**) LF in stage 4; (**d**) SFF in stage 4; (**e**) SLF in stage 4. Longitudinal section: (**f**) LF in stage 3; (**g**) SFF in stage 3; (**h**) LF in stage 4; (**i**) SFF in stage 4; (**j**) SLF in stage 4.

**Figure 4:**
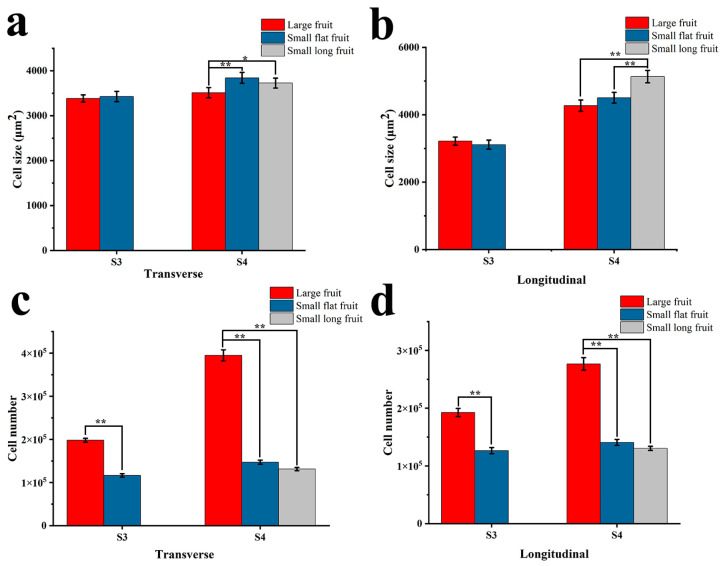
Statistics on fruit cell size and number: (**a**) cell size on the transverse section of fruit; (**b**) cell size on the longitudinal section of fruit; (**c**) cell number on the transverse section of fruit; (**d**) cell number on the longitudinal section of fruit. *: *p* < 0.05; **: *p* < 0.01.

In order to observe the difference between LF and SF cells during the crucial morphological period, the fruit tissues were sliced (Figure 3). In stage 3, there was no difference in cell size between LF and SF (Figure 3a,b,f,g and Figure 4a,b). In stage 4, the cell size of SFF and SLF on the transverse section, as well as SLF on the longitudinal section, were significantly larger than that of LF, but there is no difference between LF and SFF on the longitudinal section (Figure 3c–e,h–j and Figure 4a,b). In stages 3 and 4, the cell number of LF is significantly more than that of SF in both the transverse section and longitudinal section (Figure 4c,d). The above results showed that in the crucial morphological period, the differentiation of fruit size is not determined by cell size but by cell number.

### 2.3. Transcriptome Sequencing

To identify the mRNA expression profiles in LF and SF, 15 cDNA libraries were constructed in stage 3 (LF3-1, LF3-2 and LF3-3 for the LF; SFF3-1, SFF3-2 and SFF3-3 for the SF) and stage 4 (LF4-1, LF4-2 and LF4-3 for the LF; SFF4-1, SFF4-2, SFF4-3, SLF4-1, SLF 4-2 and SLF4-3 for the SF) using total RNA and sequenced them on the NovaSeq 6000 platform. A total of 346.70Mb clean reads were obtained after eliminating low-quality reads (Table S1). Over 85.44% of the reads were mapped to the reference *D. kaki* genome (Table S2).

**Figure 5:**
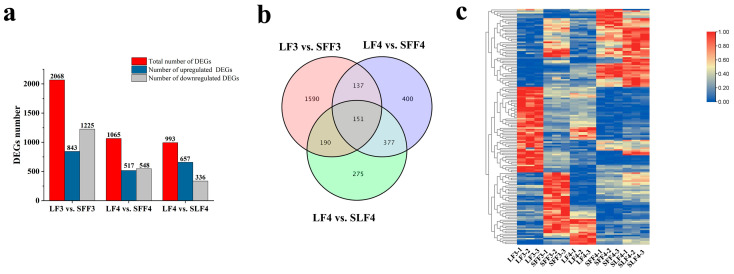
The number, Venn diagram and heat map of DEGs: (**a**) number classification of DEGs in LF3 vs. SFF3, LF4 vs. SFF4 and LF4 vs. SLF4; (**b**) Venn diagram of all DEGs; (**c**) heat map of DEGs shared by LF3 vs. SFF3, LF4 vs. SFF4 and LF4 vs. SLF4. The original expression values of the DEGs FPKM were normalized by Z-score.

The differentially expressed genes (DEGs) were compared using DESeq2 software. A total of 2068, 1065 and 993 DEGs were identified in LF3 vs. SFF3, LF4 vs. SFF4 and LF4 vs. SLF4, respectively (Figure 5a). Compared with the LF3, 843 genes were upregulated, and 1225 genes were downregulated in SFF3 (Figure 5a). Compared with the LF4, 517 genes were upregulated, and 548 genes were downregulated in SFF4; 657 genes were upregulated, and 336 genes were downregulated in SLF4, respectively (Figure 5a). In addition, a total of 151 DEGs were shared by LF3 vs. SFF3, LF4 vs. SFF4 and LF4 vs. SLF4 (Figure 5b,c). 

**Figure 6:**
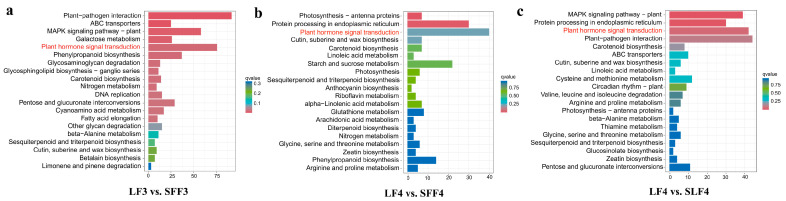
KEGG pathway enrichment analyses of DEGs. (**a**) LF3 vs. SFF3, (**b**) LF4 vs. SFF4 and (**c**) LF4 vs. SLF4.

Further analysis of DEGs was performed using the KEGG database. The plant hormone signal transduction was enriched in groups LF3 vs. SFF3, LF4 vs. SFF4 and LF4 vs. SLF4, suggesting that plant hormones play an important role in regulating persimmon fruit size (Figure 6).

### 2.4. Candidate Genes Identification 

**Figure 7:**
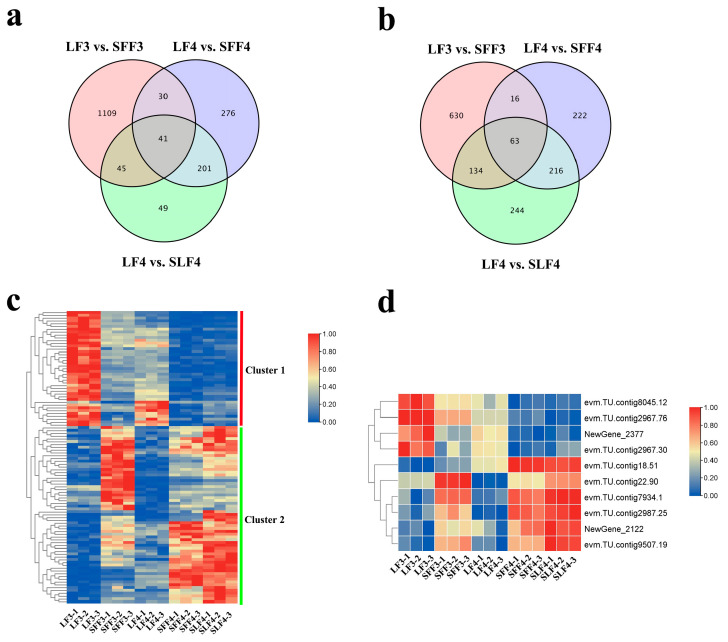
Venn diagram and heat map of DEGs: (**a**) Venn diagram of downregulated DEGs; (**b**) Venn diagram of upregulated DEGs; (**c**) heat map of co-upregulated and co-downregulated DEGs in SFF3, SFF4 and SLF4; (**d**) heat map of DEGs related to plant hormone and cell division. The original expression values of the DEGs FPKM were normalized by Z-score.

Forty-one DEGs (cluster 1) were co-downregulated, and 63 DEGs (cluster 2) were co-upregulated in SFF3, SFF4 and SLF4 (Figure 7a–c; Table S3). Among them, six DEGs related to plant hormones were identified: *ABP19A* (*NewGene_2122*), *CYP707A4* (*evm.TU.contig18.51*), *ERS1* (*evm.TU.contig22.90*) and *ERF114* (*evm.TU.contig7934.1*) were co-upregulated, and *GAI1* (*evm.TU.contig8045.12*) and *At3g51470* (*NewGene_2377*) were co-downregulated in SFF3, SFF4 and SLF4 (Figure 7d; Table S4). 

Four DEGs related to cell division were identified: *LAC17* (*evm.TU.contig9507.19*) and *CNR1* (*evm.TU.contig2987.25*) were co-upregulated, and *ANT* (*evm.TU.contig2967.76*) and *EB1C* (*evm.TU.contig2967.30*) were co-downregulated in SFF3, SFF4 and SLF4 (Figure 7d; Table S4).

### 2.5. Weighted Gene Co-Expression Network (WGCNA)

**Figure 8:**
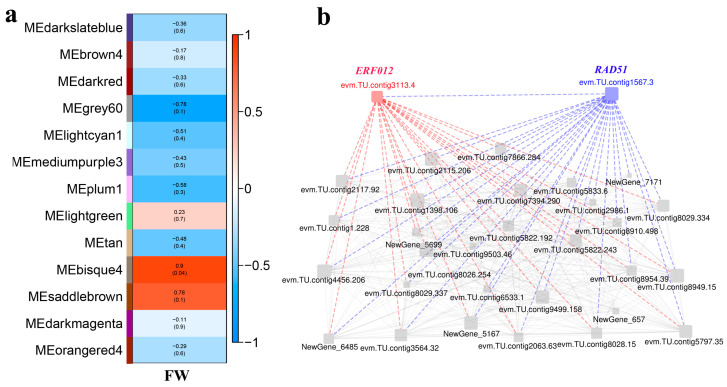
Weighted gene co-expression network: (**a**) module detection using the WGCNA; (**b**) co-expression network of genes in the bisque4 module.

To identify the gene regulatory networks associated with the persimmon fruit size, the correlation relationships between gene expression and FW were analyzed using WGCNA. A total of 26,905 genes were performed by WGCNA analysis, and 13 merged co-expression gene modules were identified. Only the bisque4 module showed a significant positive correlation with FW (Figure 8a). Further analysis revealed that 31 genes related to FW were identified in the module.

The highly connected genes of the bisque4 module were further investigated as potential key factors related to persimmon FW variation. The gene co-expression network identified two genes, *ERF012* (*evm.TU.contig3113.4*), related to ethylene signal transduction, and *RAD51* (*evm.TU.contig1567.3*), related to cell division, showing a close correlation with the FW (Figure 8b).

### 2.6. Application of Ethrel and Aminoethoxyinylglycine

**Figure 9:**
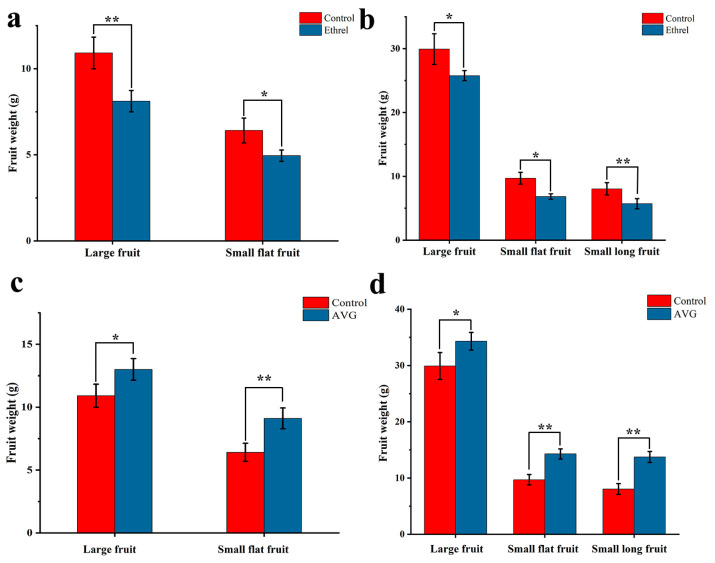
Changes in FW after application of ethrel and AVG: (**a**) changes in FW after application of ethrel in stage 3; (**b**) changes in FW after application of ethrel in stage 4; (**c**) changes in FW after application of AVG in stage 3; (**d**) changes in FW after application of AVG in stage 4. *: *p* < 0.05; **: *p* < 0.01.

Ethylene response factors, *ERS1* and *ERF114,* were identified as key candidate genes, indicating that ethylene plays an important role in persimmon fruit size formation. To verify the regulatory effect of ethylene on persimmon fruit size, we applied 100 mg/L of ethrel and 100 mg/L aminoethoxyinylglycine (AVG: ethylene inhibitor) to observe the change in FW. Compared with the control, the FW of LF and SF treated with ethrel decreased in stages 3 and 4, while the FW of LF and SF treated with AVG increased in these two stages (Figure 9). 

### 2.7. qRT-qPCR

**Figure 10:**
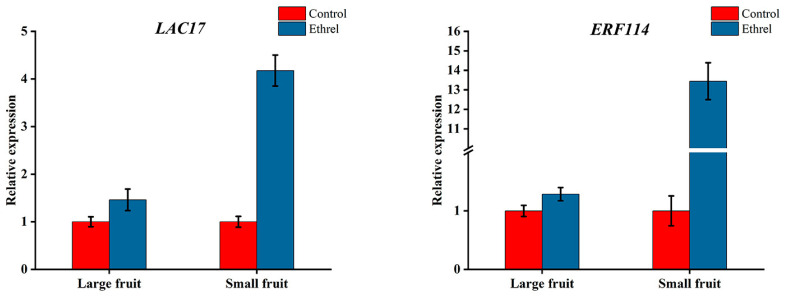
The relative expression of *LAC17* and *ERF114* after ethrel treatment.

After ethrel treatment, *LAC17* (*evm.TU.contig9507.19*) and *ERF114* (*evm.TU.contig7934.1*) were upregulated, which was consistent with the transcriptome result (Figure 10). This result not only proved the accuracy of the transcriptome data but also showed that ethylene can regulate fruit size by affecting cell division.

## 3. Discussion

The most common cultivated persimmon species is hexaploid (2n = 6x = 90), with abundant variation in fruit size [19,20]. The inconsistency in genetic background among different varieties causes interference in studying the mechanism of persimmon fruit size formation. Polygamomonoecious persimmon ‘Yaoxianwuhua’ can produce different types of fruit of different sizes, which is an ideal material for studying the regulating mechanism of persimmon fruit size. In this study, the result of morphological observation showed that stages 3–4 (June 11–June 25) are crucial periods for the differentiation of LF and SF in persimmon. 

Fruit size is influenced by the cell number or cell size. In cucumber (*Cucumis sativus*), small fruits tend to be composed of smaller cells, while some small fruits with lower cell numbers and larger cells also exist [11,21,22]. In apple (*Malus pumila*) and pineapple (*Ananas comosus*), fruit size is determined by both cell number and cell size [23,24]. In melon (*Cucumis melo*), sweet cherry (*Prunus avium*), peach (*Prunus persica*) and pear (*Pyrus spp*), fruit size was mainly influenced by cell number [25,26,27,28]. In this study, there was no difference in cell size between LF and SFF in the transverse section in stage 3 and the longitudinal section in stages 3 and 4. However, the cell size of LF was significantly smaller than that in SLF on both the transverse and longitudinal sections in stage 4 and also significantly smaller than that in SFF on a transverse section at this stage. The cell number of LF was significantly greater than that of SF on both the transverse section and longitudinal section in stages 3 and 4. These results indicate that the difference in cell number is the main reason for the differentiation of persimmon fruit size. The accumulation of cell number is conducive to the subsequent increase in persimmon fruit size.

Cell division affects fruit size by regulating the cell number. Several genes involved in cell division related to fruit size formation were found in some studies. *FW2.2*, also known as *CNR*, was the first gene identified to control fruit size. *FW2.2* influences fruit size by negatively regulating cell division in tomato [29,30]. In maize (*Zea mays*), over-expression of *ZmCNR1* and *ZmCNR2* can reduce organ and plant size by inhibiting cell division [31]. In this study, *CNR1* was downregulated in LF, suggesting that *CNR1* may promote persimmon fruit enlargement by negatively regulating cell division. *ANT,* encoding a transcription factor of the AP2-domain family, positively regulates fruit size by controlling cell division [32,33]. In this study, *ANT* was upregulated in LF, suggesting that *ANT* may promote persimmon fruit enlargement by positively regulating cell division. In addition, *AtLAC17* and *AtEB1C* were involved in regulating cell division during the growth and development of Arabidopsis [34,35]. In this study, *LAC17* and *EB1C* may be involved in regulating persimmon fruit size formation.

Ethylene often plays an inhibitory role in plant development [36]. In Arabidopsis, *EER5* can enhance ethylene reaction during signal transduction. The *eer5* ethylene hypersensitive mutant exhibits shorter siliques, curly leaves, shorter primary roots and less lateral roots [37]. In cucumber, high concentrations of ethylene can inhibit fruit elongation and cell division [12]. In apples, the application of ethylene inhibitors increased fruit size [38]. In this study, ethrel and AVG treatments resulted in a decrease and increase in fruit weight, respectively, indicating that high ethylene levels can inhibit persimmon fruit growth. As the ethylene response factors, *ERS1* and *ERF114* may play an important role in this process. After ethrel treatment, *LAC17* and *ERF114* were upregulated, indicating that ethylene can influence the persimmon fruit size by regulating cell division. The similar trend of expression of *LAC17* and *ERF114* after ethrel treatment also indicated that there might be a potential interaction between the ethylene response factor and cell division genes. 

## 4. Materials and Methods

### 4.1. Plant Material

Fruit samples of ‘Yaoxianwuhua’ persimmon (*Diospyros kaki*) were harvested in Yuanyang County, Henan Province, China (34°55′18″~34°56′27″ N, 113°46′14″~113°47′35″ E). The sampling time was 15 May 2022–15 September 2022 (0–124 days past anthesis). Samples were randomly collected every 14 days. The sample for the paraffin section is fixed in the FAA (formalin/lacial acetic acid/50% alcohol = 8:5:87). Samples for RNA extraction were immediately frozen in liquid nitrogen and stored at −80 °C.

### 4.2. Paraffin Section

The fruit samples fixed in FAA for 24h were dehydrated with a range of different concentrations of alcohol and then embedded in paraffin. After slicing and dyeing, the microslides were dried and and mounted with a cover slip. Finally, the finished microslides were observed under a light microscope (Olympus, Tokyo, Japan).

The cell size in the given images was calculated using the software ImageJ (v.1.8.0). Cell size is represented by cell area. The cell number in the transverse section = fruit transverse section area/fruit cell area in the transverse section. The cell number on the longitudinal section = fruit longitudinal section area/fruit cell area in the longitudinal section. The fruit transverse and longitudinal section areas were calculated using the software ImageJ (v.1.8.0).

### 4.3. Transcriptome

TRIzol Total RNA Isolation Kit (Sangon, Shanghai, China) was used to extract total RNA for sequencing libraries. Bioanalyzer 2100 was used to detect RNA integrity. The sequencing platform is NovaSeq 6000 (Illumina, San Diego, CA, USA). HISAT2 software (v.2.2.1) was used to match the filtered reads to the persimmon genome [19,39]. StringTie (v.2.1.1) and FeatureCounts (v.2.0.1) software were used to predict new transcripts and calculate the number of reads mapped to each gene, respectively [40,41]. Gene expression was represented by FPKM. DEseq2 (v1.18.0) was used to detect the differential mRNA expression (DEGs) (fold change ≥ 2; FDR < 0.01).

All expressed genes were used to perform weighted gene co-expression network analysis using the R package WGCNA (An R package for weighted correlation network analysis). The co-expression networks were visualized using Cytoscape (v3.8.0).

### 4.4. Ethrel and Aminoethoxyinylglycine Treatment

At dusk on 5 June 2023 (21 days past anthesis), 100 mg/L of ethrel and 100 mg/L aminoethoxyinylglycine were sprayed on the fruits of ‘YaoxianWuhua’ persimmon. The control was treated with water. Treatment was applied to three replicate trees. After one (28 days past anthesis) and three weeks (42 days past anthesis) of ethrel and aminoethoxyinylglycine treatment, fruit samples were taken and weighed.

### 4.5. qRT-qPCR

After 12 h of ethrel and water (control) treatment, fruit samples were taken for qRT-qPCR. The reaction condition: 30 s at 95 °C, 40 cycles of 3 s at 95 °C, 30 s at 60 °C. The reference gene is *GAPDH* [42]. The 2^−ΔΔCt^ method was used to calculate relative expression. The primer sequences are listed in Table S5.

## 5. Conclusion

In conclusion, stages 3–4 (June 11–June 25) are the crucial morphological periods for the differentiation of large fruit and small fruit in persimmon. In this crucial morphological period, the difference in cell number is the main reason for the differentiation of persimmon fruit size, and the accumulation of cell number is conducive to the subsequent increase in persimmon fruit size. The difference in cell number was caused by cell division. *CNR1*, *ANT, LAC17* and *EB1C*, associated with cell division, may be involved in regulating persimmon fruit size. High ethylene levels can reduce persimmon fruit size, possibly by inhibiting cell division. As the ethylene response factors, *ERS1* and *ERF114* may play an important role in this process. This study lays an empirical foundation for ongoing investigations of persimmon fruit size formation.

## Data Availability

The transcriptome sequencing raw data were deposited in the National Center for Biotechnology Information Sequence Read Archive (NCBI SRA) under the Bioproject ID PRJNA1094889 and PRJNA1116589.

## Supplementary Materials

The following supporting information can be downloaded at: https://www.mdpi.com/article/10.3390/ijms25137238/s1.
